# Malate metabolism in horticultural crops: mechanistic insights and agricultural practices for quality improvement

**DOI:** 10.1186/s43897-025-00196-6

**Published:** 2025-10-23

**Authors:** Ting-Ting Zhao, Lian-Da Du, Chu-Kun Wang, Meng-Meng Wei, Da-Gang Hu

**Affiliations:** National Research Center for Apple Engineering and Technology, Shandong Collaborative Innovation Center of Fruit & Vegetable Quality and Efficient Production, College of Horticultural Science and Engineering, Shandong Agricultural University, Tai’an, Shandong 271018 China

**Keywords:** Malate metabolism, Vacuolar transport, Acid trap, Evolutionary adaptation, Fruit quality

## Abstract

Malate metabolism bridges plant evolutionary adaptation and fruit quality regulation, serving dual roles in energy metabolism (tricarboxylic acid cycle/glycolysis) and environmental stress responses (stomatal control, pH balance). In horticulture, apple malate content dictates flavor profiles, driving divergent consumer preferences (high-sugar in Asia vs. tartness in the West), necessitating precision breeding targeting vacuolar accumulation mechanisms. Recent bioinformatic studies and transporter biology (e.g., Ma1, ALMT) have revealed genetic regulators of malate homeostasis, yet transcriptional regulation and post-translational modifications (PTMs) of transporters remain poorly understood. Notably, cultivated varieties exhibit distinct malate-related traits compared to their wild relatives, a divergence attributable to artificial selection during domestication. Additionally, agroecological factors including light, temperature, and soil conditions, dynamically regulate malate biosynthesis and storage. This metabolic plasticity reflects evolutionary adaptations influenced by domestication. This review integrates molecular physiology and domestication genetics to dissect cross-scale regulation of malate networks. We propose a transporter-engineering framework for developing market-tailored varieties and highlight unresolved questions, including PTM-mediated transporter regulation and metabolic plasticity modeling for climate-resilient crops. Bridging evolutionary adaptation with quality-driven breeding targeting malate, this synthesis advances strategies for sustainable horticulture in shifting agroclimatic landscapes.

**Supplementary Information:**

The online version contains supplementary material available at 10.1186/s43897-025-00196-6.

## Introduction

Malate, the collective term for physiologically active forms of malic acid, is ubiquitously present in nearly all plant species. This critical metabolite not only fuels plant growth through its dual role in the tricarboxylic acid (TCA) cycle and glycolytic pathway (Igamberdiev and Eprintsev 2016), but also mediates plant-environment interactions by regulating fundamental processes including stomatal dynamics (malate serves as a key osmoticum in guard cells, where its accumulation regulates turgor pressure to drive stomatal opening), aluminum ion detoxification (malate secretion chelates toxic Al^3+^ ions in the rhizosphere to form non-toxic complexes), cellular pH homeostasis (malate acts as a biochemical pH stat through its interconversion with citrate in the TCA cycle), and stress adaptation (malate maintains redox balance by modulating NAD(P)H/NAD(P)^+^ ratios under oxidative stress) (Meyer et al. 2010; Sharma et al. 2016). In horticultural applications, apple (*Malus domestica* Borkh.) stands out as a globally significant crop valued for its nutritional profile rich in minerals and vitamins. Current breeding initiatives prioritize quality enhancement, particularly regarding flavor profiles. Notably, consumer preferences exhibit geographical divergence: Asian markets represented by China demonstrate predilection for high-sugar cultivars (Zhang et al. 2023a, b), while European and North American consumers favor varieties with pronounced tartness (Svara et al. 2024). This dichotomy underscores the agricultural imperative to elucidate the molecular mechanisms governing malate biosynthesis and compartmentalization in fruit vacuoles, knowledge essential for precision breeding of market-specific apple varieties.

Recent advancements in quantitative trait loci (QTL) mapping have enabled the identification of pivotal genes modulating malate homeostasis (Gai et al. 2023; Fu et al. 2024; Oh et al. 2025). Emerging evidence highlights membrane transporter proteins as critical determinants of malate accumulation dynamics. While transcriptional regulation and post-translational modifications (PTMs)-particularly those affecting transporter protein activity-have been extensively characterized as key regulators of plant developmental processes (Agbemafle et al. 2023; Yi and Shan 2023), the mechanistic interplay between these modifications and malate metabolic networks remains unresolved.

The biosynthesis and vacuolar sequestration of malate in fruit exhibit pronounced sensitivity to agroecological conditions, with photoperiodic regulation, thermal fluctuations, soil moisture, and edaphic factors serving as primary determinants (Oliveira et al. 2023; Zheng et al. 2023a). This metabolic plasticity manifests through dynamic adjustments in malate concentrations—a principal organic acid mediator of plant environmental response (Xie et al. 2023; Yu et al. 2025). Furthermore, the whole genomic analyses reveal substantial divergence in malate profiles between domesticated cultivars and their wild progenitors, an evolutionary consequence of multigenerational selection pressures combining artificial hybridization with sustained anthropogenic cultivation regimes (Yu et al. 2021a, b; Zhang et al. 2023a, b).

This systematic review pursues two synergistic objectives: (1) to establish a novel cross-scale analytical paradigm integrating molecular physiology with domestication genetics, and (2) to delineate evolutionary selection pressures shaping malate metabolic pathways across plant lineages. Building upon emergent insights into malate homeostasis regulation, we critically evaluate crucial determinants governing vacuolar malate accumulation dynamics from genome reconstruction to xylem loading efficiency, thereby identifying knowledge gaps requiring prioritized investigation. Furthermore, we propose an operational framework for developing trait-specific genetic repositories through transporter protein engineering.

## Malate dynamics in plant cells: biosynthesis, membrane transport, and vacuolar sequestration mechanisms

Malate plays crucial roles in plant metabolism. The synthesis and degradation of malate occur in multiple organelles via glycolysis (Dong et al. 2018), glyoxylate cycle (Wu et al. 2020) and TCA cycle (Liu et al. 2023). Malate can also be converted into pyruvate by malic enzyme, linking it to other metabolic processes such as gluconeogenesis or amino acid synthesis (Zhang et al. 2022a). The accumulation of malate in cells mainly depends on three factors: synthesis, degradation and storage in vacuole by a series of transporter proteins and proton pumps (Sunita et al. 2018; Zhang et al. 2022b). This section primarily discusses the various pathways involved in the metabolism and transport of malate, as well as its sequestration in the vacuole.

### The complex biosynthetic pathways of malate in the cytoplasm

**Fig. 1:**
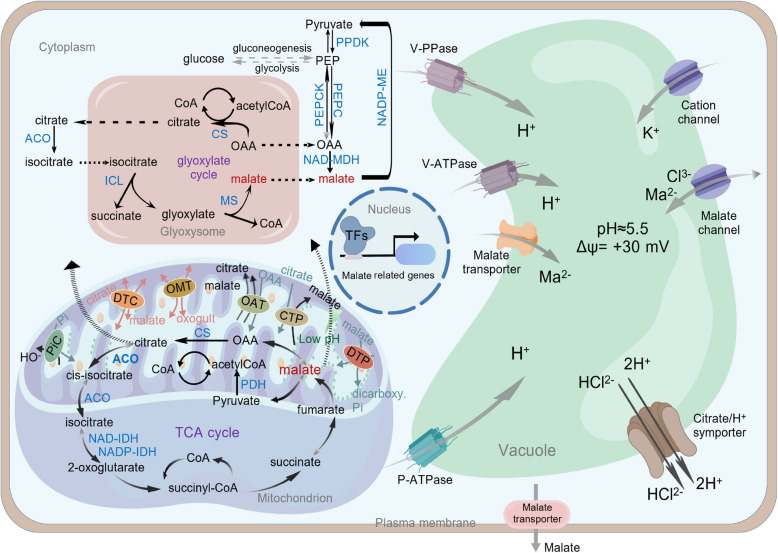
The metabolism and transport of malate in plant cells. The key enzymes listed in the figure include: PEPC, phosphoenolpyruvate carboxylase; PEPCK, phosphoenolpyruvate carboxykinase; NAD-MDH, NAD-malate dehydrogenase; NADP-ME, NADP-malic enzyme; CS, citrate synthase; ACO, aconitase; IDH, isocitrate dehydrogenase; NAD-ME, NAD-malic enzyme. A bidirectional arrow indicates a reversible reaction

Fruit acidity depends on the accumulation of organic acids, which is highly controled by its metabolism and transport. Malate is the most important organic acid in apples, contributing 90% of the total organic acids in mature fruit (Ma et al. 2015). Phosphoenolpyruvate (PEP) is converted into malate through a two-step process catalyzed by phosphoenolpyruvate carboxylase (PEPC) and cytosolic NAD-dependent malate dehydrogenase (NAD-cyMDH) (Setien et al. 2014; Singh et al. 2022). PEPC has been reported to fix CO₂ (HCO_3_^−^) using PEP as a co-substrate to catalyze the formation of oxaloacetate (OAA). OAA are subsequently converted to malate through MDH (Shi et al. 2015; Han et al. 2018). The functions of *PEPC* and *MDH* have been characterized in apple (Yao et al. 2009, 2010). Overexpression of apple *NAD-cyMDH* leads to an increase in malate content (Yao et al. 2010; Zhang et al. 2022b). Moreover, *MDH12* in mitochondria has been shown to positively regulate malate content in apple calli (Gao et al. 2022). Malate content exhibits dynamic changes during fruit development, with a sharp increase observed at early developmental stage, reaching its peak by the end of cell division phase (Etienne et al. 2013). The transcription level of certain *PEPCs* increase during the early stages of fruit growth and development, while several *MDHs* also exhibit high expression at this stage. For example: 1-methylcyclopropene (1-MCP) treatment enhances malate content through simultaneous upregulating *MdPEPC*/*MdcyMDH* expression in apple fruit (Liu et al. 2016). And overexpression of *MdcyMDH* results in increased malate accumulation in both apple calli and tomato (Yao et al. 2009). These expression patterns are consistent with the roles of *PEPC* and *MDH* in malate biosynthesis during early fruit development (Yao et al. 2009, 2010). As apple fruit matures, malate gradually decreases with the upregulation of NADP-dependent malic enzyme (NADP-ME) and downregulation of *PEPC* (Shi et al. 2015). In Arabidopsis (*Arabidopsis thaliana*) proteome, there are a total of four NADP-malic enzymes (AtNADP-ME1, ME2, ME3, and ME4), among which NADP-ME4 exhibits the highest conversion efficiency toward malate (Wheeler et al. 2005). Research on 42 F_1_ progenies of Gala and Xiahongrou, with 20 other apple cultivars reveals that the expression level of *MdNADP-ME* is correlated with malate content (Fu et al. 2024). Moreover, in berries, malate is decarboxylated into pyruvate by NADP-ME under photosynthetic conditions (Sweetman et al. 2009). In summary, malate metabolism mainly relies on two key enzymes, NAD-cyMDH and PEPC. The glycolysis product PEP is carboxylated into OAA by PEPC, and OAA in then reduced to malate by NAD-cyMDH in cytoplasm (Fig. 1).

### Malate accumulates through shuttling across various membrane systems

The accumulation of malate within cells is governed by two key processes: malate efflux (Maruyama et al. 2019; Yamada et al. 2025) and malate influx namely vacuole sequestration. Unlike animal cells, plant cells possess large central vacuoles occupying up to 90% of the cell volume, which serve as reservoirs for organic acids (Karcz and Burdach 2024). This compartmentalization requires the synergetic action of vacuolar proton pumps and tonoplast localized malate transporters. Vacuolar proton pumps, such as V-ATPase, V-PPase and P-type ATPase, acidify the vacuole by transporting protons into vacuole across the vacuolar membrane (Liang et al. 2020; Cosse and Seidel 2021). The acidified vacuolar environment enhances the activation of malate once they are transported into the vacuole. This activation helps maintain an electrochemical gradient across the vacuolar membrane, driving a continuous malate influx, a process widely recognized as the "acid trap" (Krebs et al. 2010; Kriegel et al. 2015).

In apple, the transport of malate to vacuoles is achieved through a serials of transport proteins and proton pumps such as aluminum-activated malate transporter 9 (Ma1/MdALMT9) (Wang et al. 2023; Hu et al. 2025), vacuolar ATPase subunit A (MdVHA) (Hu et al. 2017; Zhang et al. 2020a, b) and vacuolar pyrophosphatase (MdVHP) (Xiang et al. 2024). In tomato (*Solanum lycopersicum*), SlALMT9 has an effect on malate content in tomato fruit to a large extent (Ye et al. 2017; Liu et al. 2024b). Interestingly, a plant-specific tonoplast dicarboxylate transporter (tDT) from the solute carrier family 13 (SLC13) family posesses dual functionality of transporting malate into the vacuole and exporting citrate to cytoplasm in Arabidopsis (Frei et al. 2018). Malate undergoes rapid oxidation through sequential reactions catalyzed by mitochondrial malate dehydrogenase (mMDH) and NAD-dependent malic enzyme (NAD-ME), OAA and pyruvate, which were subsequently condensed to form citrate in the mitochondrial matrix (Zhang et al. 2022a, b). This citrate efflux acidifies both mitochondrial and vacuolar compartments, triggering a pH-regulatory response mediated by two key bidirectional transporters: (1) dicarboxylate carrier 2 (DIC2) at the mitochondrial membrane (Zhang et al. 2022a, b), and (2) tDT at tonoplast (Frei et al. 2018). These transporters coordinately stabilize cellular pH by coupling citrate export and malate import. Moreover, the expression of *tDT* is induced by malate (Vera et al. 2003). Genetic studies on apple fruit suggest that *MdtDT* has a negative role in citrate accumulation (Liao et al. 2021). Similarly, in navel orange (*Citrus sinensis* cv Washington), the AttDT homolog citrate transporter 1 (CsCit1) functions as a citrate transporter that mediates citrate efflux from the vacuole (Shimada et al. 2006).

On the other hand, cytosolic malate content is modulated by plasma membrane efflux transporters. In acidic soils, aluminum ions (Al^3+^) toxicity severely restricts crop productivity. To alleviate this stress, plants release dicarboxylate or tricarboxylate chelators from their roots to reduce Al^3+^ toxicity in the rhizosphere (Kar et al. 2021). Malate, a key dicarboxylate, acts as a metal chelator upon cytoplasmic entry of Al^3+^ or other toxic ions, facilitating their transport into vacuoles and thereby alleviating cellular toxicity (Sasaki et al. 2022; Sun et al. 2022). Many plants resisted toxicity by similar evolving mechanisms (Badia et al. 2020; Luo et al. 2024; Li et al. 2025).

## Multi-tiered regulatory architecture of malate: transcriptional circuits, RNA splicing dynamics, and post-translational landscapes

### Transcriptional circuits in malate metabolism and transport

**Fig. 2:**
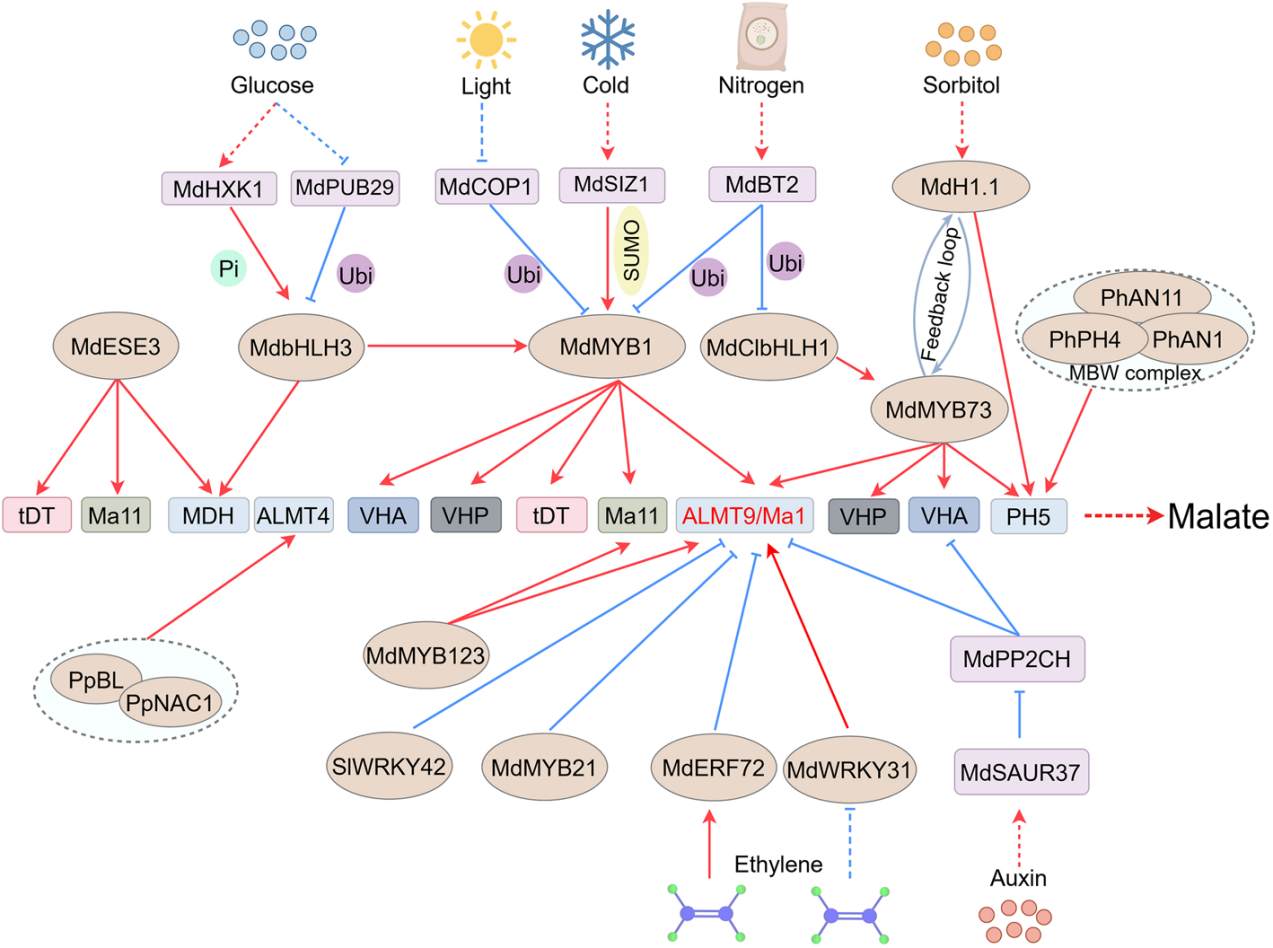
Regulation of complex malate synthesis and transport. In the figure, MBW represents the MYB-bHLH-WD40 complex. The figure only presents the well-studied regulatory pathways. Arrows represent regulatory relationships, with corresponding post-translational modifications (e.g., phosphorylation, ubiquitination, SUMOylation) annotated on either side of the arrows

The accumulation of malate is predominantly governed by its metabolism and transport, and the transcriptional regulation of these two biological processes has been extensively studied. In apple, the basic helix-loop-helix (bHLH) transcription factor MdbHLH3 upregulates the *MdcyMDH* expression by binding to its promoter directly, thereby promoting malate biosynthesis and accumulation (Yu et al. 2021a, b). The transcriptional regulation of malate transporters and proton pumps is also orchestrated by a sophisticated regulatory network involving multiple transcription factor families such as MYB (Huang et al. 2023), bHLH (Liu et al. 2022), WRKY (Yang et al. 2023), and ethylene response factor (ERF) (Xu et al. 2024a). And apple is the most extensively studied species related to malate metabolism and transport. In apple, Ma1, a member of the ALMT family, has been identified as a determinant of vacuolar malate storage (Li et al. 2020, 2024). The transcription of *Ma1* is regulated by multiple transcription factors including the WRKY transcription factor 31 (MdWRKY31)–MdERF72 network, the R2R3-MYB transcription factor 73 (MdMYB73), and MdbHLH3 (Wang et al. 2023). Interestingly, sorbitol-induced linker histone MdH1.1 functions as a transcription factor that directly binds to the promoters of *MdMYB73*, *MdCIbHLH1*, and *MdPH5* (*P-type ATPase*) to coordinately enhance the expression of *Ma1* and *MdPH5*, thereby regulating malate accumulation (Hu et al. 2025). Furthermore, MdMYB73 interacts with MdClbHLH1 to co-activate the expression of *Ma1*, *vacuolar ATPase subunit A* (*MdVHA-A)* and *vacuolar pyrophosphatase 1* (*MdVHP1*) (Hu et al. 2017). The MdMYB1/10-MdbHLH3 complex modulates mutiple proton pumps, including *MdVHA-Bs*, *MdVHA-E2*, *MdVHP1*, as well as the tranporter encoding gene *MdtDT*, thereby enhancing vacuolar acidification (Hu et al. 2016a). In addition, the ERF transcription factor MdESE3 transcriptionally activates *MdMDH12*, *MdtDT*, and *Ma11*, leading to increased malate synthesis and transport to vacuole (Zheng et al. 2024). MdMYB123 simultaneously transcriptionally activate the expression of two functional genes, *Ma1* and *Ma11*, thereby promoting malate accumulation (Zheng et al. 2023b). In peach (*Prunus persica*), the NAM-ATAF1/2-CUC2 (NAC) members PpBL-PpNAC1 heterodimer activates the transcription of *PpALMT4* to increase malate content (Chen et al. 2025). In petunia (*Petunia atkinsiana*), the PH4 (MYB)-AN1 (bHLH)-AN11 (WD40) MBW ternary complex directly upregulates the transcriptional level of *PH5*, thereby affecting malate dynamics (Fig. 2; Verweij et al. 2016).

In addition to the transcriptional activation regulation mentioned above, there are also some transcription factors that act as repressors in malate accumulation. For example, MdMYB21 suppresses *Ma1* expression and malate accumulation by binding to its promoter (Peng et al. 2023). The tomato (*Solanum lycopersicum*) SlWRKY42 directly downregulates the expression of *SlALMT9*, leading to a reduction in malate content (Ye et al. 2017). A similar mechanism has been proposed to account for the action of *AtALMT1* (Ding et al. 2013)*.* Taken together, the transcriptional regulation of malate metabolism and transport is a precise and sophisticated network.

### RNA splicing dynamics in malate metabolism and transport

Alternative splicing (AS) is a key post-transcriptional regulatory mechanism that shapes plant phenotype and metabolic adaptation (Dwivedi et al. 2023; Sun et al. 2024). Through evolutionary selection of splice variants, plants generate transcriptomic diversity to optimize environmental responses. As an organic acid critical for stress adaption, emerging evidence suggests that AS also modulates malate metabolism and transport. The *Ma1* gene encodes two functionally distinct isoforms, Ma1α and Ma1β, through alternative splicing. These isoforms exhibit dynamic dimerization behavior, forming both Ma1α homodimers and Ma1α-Ma1β heterodimers. Under the condition of Ma1β deficiency, Ma1α predominantly self-associates as homodimers. However, increasing Ma1β levels competitively displace this equilibrium, preferentially promoting heterodimer formation. Notably, when the *M*a1β:Ma1α ratio reaches a critical threshold of ≥ 1:8, the isoforms demonstrate synergistic cooperation that significantly enhances malate transport activity (Li et al. 2024). In *Chlamydomonas reinhardtii,* alternative splicing generates two *phosphoenolpyruvate carboxykinase* (*PEPCK*) isoforms namely *ChlrePEPCK1* and *ChlrePEPCK2*, which display differential malate response. *ChlrePEPCK1* is activated by malate to promote malate accumulation, whereas *ChlrePEPCK2* exhibits a weaker response to malate (Torresi et al. 2023).

### Post-translational landscapes in malate metabolism and transport

In addition to transcriptional regulation, PTMs also play crucial roles in malate metabolism and transport, including phosphorylation/dephosphorylation, ubiquitination and SUMOylation (Boxall et al. 2017; Caburatan and Park 2021). These PMTs target key catalytic enzyme (Hartman et al. 2023), transcription factors (Gao and Dubos 2024), or transporter proteins (Xu and Wang 2021), highlighting their broad regulatory impact.

During malate synthesis through gluconeogenesis and the glycolate pathway, PEPCK phosphorylates the serine residues of PEPC to enhance malate synthesis (Zhan et al. 2023). There has been also some research on the phosphorylation of membrane transport proteins by protein kinases. MdPP2CH*,* a member of the protein phosphatase 2CH subfamily, dephosphorylates and degrades members of the H^+^-ATPase family (MdVHA-A3*,* MdVHA-B2*,* MdVHA-D2), reducing malate accumulation (Jia et al. 2018). *SAURs* can be rapidly induced within minutes by active auxin (Franco et al. 1990). MdSAUR37 interacts with MdPP2CH and suppresses its phosphatase activity, thereby modulating malate accumulation (Fig. 2; Jia et al. 2018). Meanwhile, auxin-responsive MdARF2 transcriptionally represses *MdMATEL1* and *MdcyMDH*, which are involved in the synthesis and transport of malate (Wang et al. 2025a).

In addition to the modifications of aforementioned synthases and transporters, PTMs of upstream transcription factors are even more prevalent. In apple, extensive research has focused on PTMs of the MYB-bHLH-WD40 (MBW) complex (Liu et al. 2019; Fan et al. 2022; Wang et al. 2024a, b). Light induces the expression of *constitutively photomorphogenic 1* (*MdCOP1*), which encodes an E3 ubiquitin ligase. MdCOP1 ubiquitinates MdMYB1 to promote the degradation of the latter, leading to reduced malate accumulation in the cell (Li et al. 2012). Similarly, under high nitrate conditions, the BTB-BACK-TAZ scaffold protein MdBT2 ubiquitinates MdCIbHLH1, disrupting its interaction with MdMYB73 and downregulating malate content (Zhang et al. 2020a, b). Furthermore, the glucose sensor hexokinase 1 (MdHXK1) interacts with MdbHLH3, resulting in its phosphorylation and enhanced activity. At the same time, glucose suppresses the expression of *Plant U-box E3 ubiquitin Ligase 29* (*MdPUB29*), whose encoding product acts as an E3 ubiquitin ligase to ubiquitinate MdbHLH3. Taken together, glucose enhance the activity and accumulation of MdbHLH3 by promoting its phosphorylation and inhibiting its ubiquitination, respectively. Ultimately, these modifications facilitate the transcriptional activation activity of MdbHLH3 on target genes, resulting in the accumulation of malate (Hu et al. 2016b, 2019). Additionally, low temperature promotes sap and miz1 domain containing ligase1 (MdSIZ1), a SIZ/PIAS-type SUMO E3 ligase, mediated SUMOylation of MdMYB1, stabilizing MdMYB1 and enhancing malate transport and accumulation in vacuole (Fig. 2; Zhou et al. 2017).

### Epigenetic regulation of malate metabolism

Epigenetics plays a crucial role in regulating malic acid metabolism by influencing gene expression through DNA methylation (Liu et al. 2024a), histone modifications (Wang et al. 2025b), and non-coding RNAs (Nakul et al. 2024). Methylation typically occurs on DNA (cytosine residues) (Yu et al. 2023; Kumar and Mohapatra 2021) or histones (lysine or arginine residues) (Ling et al. 2022). In apples, histone methylation has been shown to regulate the expression of malic acid biosynthesis genes during fruit development and ripening (Zheng et al. 2024). Specifically, trimethylation of histone H3 at lysine 4 (H3K4me3) is associated with the activation of malic acid-related genes (Chen et al. 2022), while trimethylation at lysine 27 (H3K27me3) is linked to their repression (Zhao et al. 2024). These epigenetic modifications provide a layer of regulatory control that integrates environmental and developmental signals to fine-tune malic acid levels.

## Phylogenomic signatures of malate domestication: malic acid trait selection in horticultural crops and *ALMT* family expansion in apple

### Targeted selection of malate traits in horticultural varieties

Consumer preferences make acidity a crucial factor in the genetic improvement of fruit quality. Recent studies have revealed a strong correlation between genomic variations and quality traits in apple fruit (Lin et al. 2023). Notably, significant genomic variations between cultivated apples and their wild relatives is largely attributed to artificial domestication. Similar high-throughput sequencing efforts have been applied to other perennial fruit crops such as pear (*Pyrus pyrifolia*) (Shirasawa et al. 2021), peach (Oncel and Ozkilinc 2025), and grape (*Vitis vinifera*) (Xiao et al. 2023), to investigate their genetics and domestication processes. Intriguingly, across these cultivated species, the content of malate appears to a key targeted trait under selection.

Horticultural crops exhibit significant variation in organic acid content across different families and genera, both in terms of composition and relative abundance. As discussed above, malic acid is the predominant organic acid in apple (*Rosaceae*), while citric acid dominates in citrus (*Rutaceae*). Notably, even within the same family or genus, distinct cultivars exhibit significant difference in organic acids. For example, in peach, white-fleshed cultivars typically accumulate higher levels of malic acid, whereas yellow-fleshed cultivars are richer in citric acid (Zheng et al. 2021). The organic acid composition in pear is similar to that of apple, with malic acid being the most abundant, followed by citric acid (Akagic et al. 2022). However, the overall level of malic acid in pear is relatively lower than that of apple. In contrast, grape exhibits a more diverse organic acid profile, including tartaric acid, malic acid, citric acid, oxalic acid, and quinic acid (Wang et al. 2025c). In grape, the content of malic acid continuously increases during immature stage and gradually decreases as the fruit ripens (Famiani et al. 2005). And citric acid is only detectable in immature grape fruit (Vicol and Duca 2023). Recent advances in breeding research have identified key genes regulating malic acid variation through bioinformatics analysis, providing valuable potential targets for genetic improvement (Zhang et al. 2015; Verta et al. 2016; Zhou et al. 2021; Bagwell et al. 2023). For instance, a genome-wide association study (GWAS) in tomato identifies a single nucleotide polymorphism (SNP) on chromosome 6 linked to malate content, designated as *tomato fruit MaLATE on chromosome 6* (*TFM6*) (Ye et al. 2017). Additionally, a 3 bp InDel insertion in *SlALMT9* promoter is shown to attenuate the repressive effect of of SlWRKY42 on *SlALMT9*, leading to increased malate accumulation (Ye et al. 2017).

### The *ALMT* family in apple has undergone genetic expansion

**Fig. 3:**
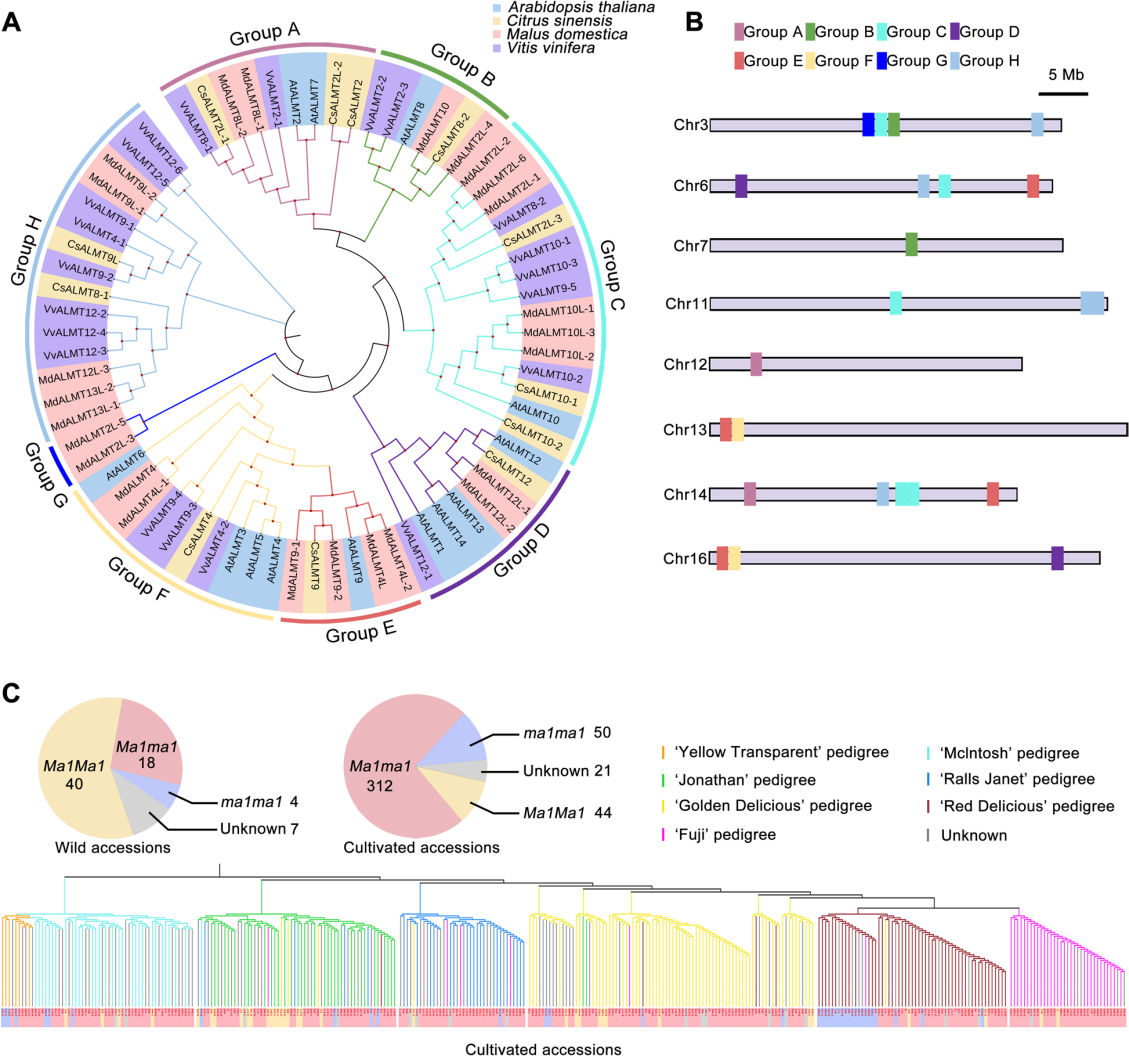
Phylogeny of *ALMTs* across four species; *ALMT* gene distribution and *Ma1* evolution in apple. **A**. Phylogenetic tree of ALMTs in *Malus domestica*, *Citrus sinensis*, *Vitis vinifera* and *Arabidopsis thaliana*. The ALMTs are divided into eight groups (Group A-H). The phylogenetic analysis based on the C_LUSTAL_ W protein alignment, which was constructed using the neighbor-joining method with a bootstrap of 1000 iterations*.* **B**. Genomic distribution of *ALMTs* in *Malus domestica*. **C**. Distribution of *Ma1* genotypes in wild and different cultivated apple accessions. Detailed information including the genome, genome annotation are recorded in Table S2

ALMTs play crucial roles in transmembrane transport of malate (Pradhan and Datta 2021; Qin et al. 2022; Peng et al. 2024; Rahman et al. 2024). In apple, Ma1 has been specifically characterized as a key transporter of malate accumulation (Wang et al. 2023; Gao et al. 2024; Li et al. 2024). To investigate the genetic and evolutionary relationships of malic acid across different species, we conducted a comprehensive phylogenetic analysis of ALMT protein family from four representative species with varying malate contents: sweet orange (*Citrus sinensis*) (low), grape (moderate), apple (high), and the model plant Arabidopsis (Fig. 3A). Overall, there are 12 *ALMTs* in arabidopsis, 12 *ALMTs* in sweet orange, 21 *ALMTs* in grape, and 25 *ALMTs* in apple, respectively. The largest *ALMT* family among the four species indicates a gene family expansion of *ALMT*s in apple (Table. S1). Using Arabidopsis as a reference, ALMTs in these four species were divided into eight groups (Groups A-H). The results based on phylogenetic analysis indicate that ALMTs are generally evenly distributed across most species; however, certain species exhibit notable exceptions. In Group H, ALMTs from Arabidopsis are absent (Table. S1). Interestingly, as a species renowned for its high malic acid content in fruit, apple ALMTs are distributed in all eight subfamilies and display a unique evolutionary lineage of Group G, which may contribute to its specific organic acid profile especially rich in malic acid. Grape ALMTs represent the second largest family among these four species but show no representatives in Groups E and G (Table. S1). This absence May contribute to the intermediate Malic acid accumulation observed in grape. In contrast, sweet orange contains only 12 ALMTs, a quantity comparable to that in Arabidopsis, which may account for its low malic acid content.

According to previous studies, the chromosomal distribution of genes significantly influences their function (Yang et al. 2011; Xing et al. 2025). To explore the distribution of *ALMTs* in apple, we mapped these genes to chromosomes. Intriguingly, the distribution was quite uneven across chromosomes, with notable enrichment on chromosomes 3, 6, and 14. Notably, chromosome 14 harbored the highest number of *ALMT*s, with a total of five genes. Among these, the four members of apple-unique Group E were distributed on chromosomes 6, 13, 14, and 16 (Fig. 3B). Taken together, the expansion of *ALMTs* in apple and their non-random chromosomal distribution may contribute to its characteristically high malate content.

### Artificial selection of *Ma1* during the evolution from wild to cultivated species

Cultivated apple varieties are derived from two wild progenitors, *Malus sieversii* (*M. sieversii*) from the Tianshan region and *Malus sylvestris* (*M. sylvestris*) from Europe, both contributing important domestication traits (Sun et al. 2020). Studies indicate that fruit from Tianshan region are larger, with a diameter of around 6 cm, and contain lower malate levels compared to other wild relative (Davies et al. 2022), which are consistent with the high morphological and genetic similarity observed between *M. sieversii* and cultivated varieties (Shan et al. 2021). It is interesting that the major QTL of malate, *Ma1*, has undergone purposeful artificial selection during evolution. A naturally occurring point mutation (G to A) at base position 1455 within *Ma1* results in a truncated, non-functional allele (*ma1*), giving rise to three distinct genotypes at the Ma locus: *Ma1Ma1*, *Ma1ma1*, and *ma1ma1* (Bai et al. 2012). The frequency of the ‘A’ allele is relatively low in *M. sylvestris* (4.5%), but considerably higher in *M. sieversii* (33.3%) and cultivated apple (55.6%) (Duan et al. 2017; Sun et al. 2020). The domestication of apple from its wild ancestors involved a notable reduction in fruit organic acid levels, a change likely favored by evolution to enhance edibility and thereby promote more efficient seed dispersal by animals and humans (Duan et al. 2017; Liao et al. 2021). For example, wild apple species (e.g., *Malus sieversii*) typically accumulate significantly higher levels of malate than domesticated apples (*Malus domestica*), with concentrations averaging 2.2-fold greater; this elevated acidity contributes to their characteristically sour taste—a trait likely diminished during domestication through human selection for sweeter, lower-acidity cultivars (Ma et al. 2015). Analysis of published resequencing data (Liao et al. 2021) from 70 wild and 427 cultivated apple accessions at the *Ma1* locus SNP (Ma locus-1455A; Chr16:3179027) reveal a striking shift in genotype frequencies during domestication. In wild apples, the distribution of *Ma1Ma1:Ma1ma1:ma1ma1* was 40:18:4, with the high-acid *Ma1Ma1* genotype accounting for 57.1%. In contrast, cultivated apples exhibited a distribution of 44:312:50, with the frequency of *Ma1Ma1* reduced to 10.3% (Fig. 3C). The pronounced reduction of the high-acidity *Ma1Ma1* genotype, alongside a marked enrichment of the low-acidity *ma1ma1* genotype, has led to the displacement of *Ma1Ma1* as the predominant allele in cultivated apples (Fig. 3C)—a genetic shift that mirrors the decline in fruit acidity during domestication. These findings indicate that selection at the *Ma1* locus likely contributed to the evolution of reduced acidity in cultivated apples, underscoring a potential role for *Ma1* in the transition from wild to domesticated forms.

Overall, malate plays an important role in the genetic regulation and domestication process of plants. Its genetic regulation can provide a theoretical foundation for the improvement of fruit quality in horticultural crops and molecular breeding.

## Agro-environmental modulation of malate biosynthesis: cultivation-driven dynamics in horticultural crops

Building upon the established genetic foundations of malate homeostasis in horticultural species (as systematically examined in preceding sections), we now interrogate the agroecosystem dynamics governing malate accumulation in commercially valuable crops. As primary determinants of fruit metabolic profiles, cultivation protocols–encompassing cultural interventions (rotational systems, canopy management), edaphic regulation strategies (precision irrigation, conservation tillage), and nutrient management protocols (biofertilizer integration, mineral supplementation)-exert profound phytochemical modulation through microenvironmental recalibration. These cultivation strategies collectively modulate critical growth parameters including photosynthetic efficiency, rhizosphere biogeochemistry, and thermal stress responses, thereby establishing an environmental metabolomic continuum. This section provides a mechanistic analysis of how such anthropogenic cultivation pressures interface with plant metabolic networks to shape malate accumulation patterns.

### Regulating malate accumulation in crops: the role of management practices in modulating biosynthesis and vacuolar storage

**Fig. 4:**
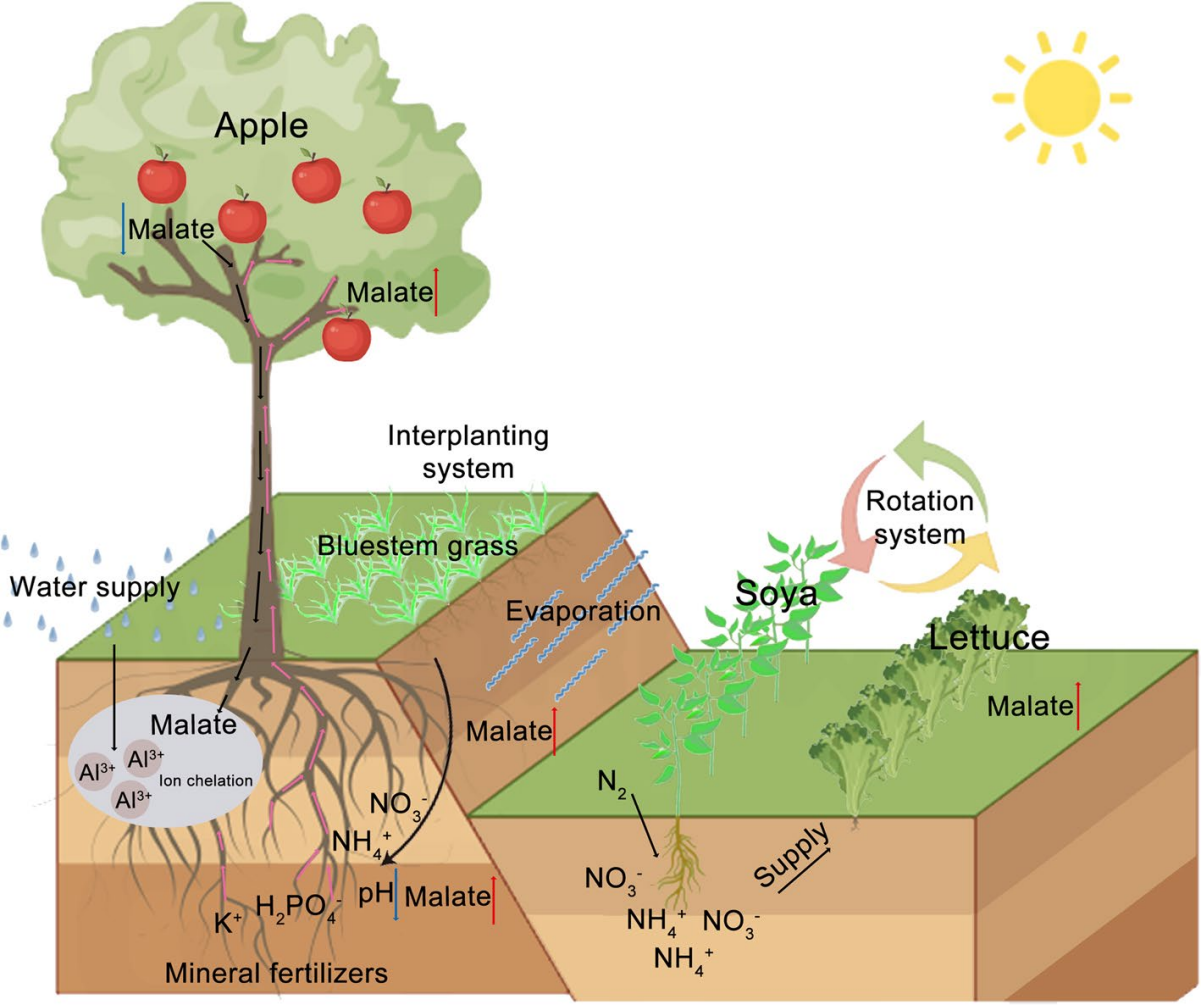
Schematic diagram of the effects of agronomic practices on malate levels in horticultural crops. Excessive water irrigation leads to soil acidification and the accumulation of toxic aluminum ions (Al^3+^). In response, apple tree roots secrete malate, which chelates Al^3+^ in acidic soils, thereby reducing its toxicity. The interplanting system of apple and bluestem grass, together with the application of mineral fertilizers supplying essential nutrients such as K^+^, H₂PO₄^−^, NO₃^−^, and NH₄^+^, helps to reduce water evaporation, stabilize pH fluctuations of soil, and ultimately promote malate accumulation in the above-ground parts of the apple tree. In the crop rotation system, soybean contributes to biological nitrogen fixation, enriching the soil with nitrogen and providing a more fertile environment for the subsequent lettuce crop

Soil nutrient composition and rhizosphere microenvironments critically influence crop growth and development, driving agricultural emphasis on strategic soil management to optimize nutrient profiles. Empirical evidence identifies crop rotation as the most effective practice for modulating soil nitrogen dynamics (Liu et al. 2023). Nitrate-form nitrogen stimulates MdBT2-mediated ubiquitination degradation of related transcription factors in apples, consequently suppressing malate biosynthesis. Annual crop systems frequently employ legume-cash crop rotations, notably legume-lettuce sequences (Fig. 4), leveraging rhizobial nitrogen fixation (Wang et al. 2024a, b) to enhance lettuce nitrogen assimilation. This dual-phase strategy reduces nitrate toxicity while paradoxically elevating malate concentrations, thereby improving flavor profiles. In perennial arboriculture, legume intercropping capitalizes on root nodule symbiosis to supply bioavailable nitrogen (Wang et al. 2025d), with optimized nitrogen levels increasing apple leaf photosynthetic efficiency by 18–24% (Xu et al. 2024b). Vineyard intercropping trials demonstrate legumes elevate fruit sugar-acid ratios by 48.52–88.56% while reducing organic acids by 21.55–31.26% (Pornaro et al. 2022). Nitrogen-deficient conditions induce the conversion of phloem-derived aspartate to xylem-mobile malate, functioning as an osmotic regulator in soybeans (Vitor et al. 2018).

Additionally, standard orchard management practices incorporate cover cropping and formative pruning to optimize tree growth conditions. Research demonstrates that interplanting Schizachyrium scoparium (little bluestem grass) among young apple trees reduces soil pH by 6.83%−7.19%, establishing a mildly acidic microenvironment conducive to tree development and enhancing foliar malate accumulation (Schwinning et al. 2017). Cover cropping further improves root zone moisture retention, mitigates soil compaction and hydraulic losses, and subtly augments soil nutrient availability.

Formative pruning remains a cornerstone of orchard management, with five primary tree architectures recognized: Bush, Cordon, Espalier, Fan, and Step-over espaliers (Zhang et al. 2021). Pomaceous species such as apples and pears thrive under Cordon and Espalier configurations, while stone fruit like cherries exhibit superior performance in Fan systems. These structured forms enhance inter-branch photosynthetic efficiency, stimulate carbon metabolism, and promote sugar-acid compound accumulation (Matias et al. 2023).

In summary, agricultural interventions including crop rotation, intercropping, orchard cover cropping, and formative pruning have critical effect on malate accumulation by inducing specific metabolic pathways, as systematically illustrated in Fig. 4.

### Water and soil management practices: their impact on malate accumulation dynamics in agricultural systems

Under agricultural management systems, strategic water supplementation proves critical for crop cultivation, necessitating coordinated irrigation-tillage implementation. Hydration regimes and tillage operations fundamentally modify soil architecture. In sandy substrates, rapid water loss through evaporation necessitates tillage-mediated capillary channel disruption between sand particles to enhance water retention (Honsdorf et al. 2022). Conversely, clay soils with reduced pore dimensions exhibit post-irrigation water stagnation, impeding both evaporation and plant uptake while fostering anaerobic conditions conducive to phytotoxin formation. These contrasting mechanisms underscore the imperative for soil-specific irrigation-tillage adaptations in orchard management.

Apple phenological studies reveal intensive leaf energy metabolism and respiratory activity during fruit expansion phases, driving substantial diurnal water consumption (Duan et al. 2017). Concurrently, malate concentrations progressively accumulate to peak levels. Upon transitioning to maturation stages, fruit quality parameters shift as malate content declines (Han et al. 2025), coinciding with reduced root hydraulic demand. Excessive irrigation during this phase elevates malate retention while suppressing sugar accumulation (Tao et al. 2023), thereby compromising sensory quality. Historical cultivation patterns demonstrate hillside-grown apples under water-limited conditions develop enhanced sugar-acid profiles and flavor complexity (Jin et al. 2018).

Controlled trials confirm water restriction elevates malate and citrate concentrations in lettuce irrespective of nitrogen availability (Ncama and Sithole 2022). Prolonged over-irrigation induces soil acidification and Al^3+^ accumulation, which triggers root malate efflux and vacuolar depletion through previously described mechanisms. Collectively, water-tillage regimes significantly influence fruit malate dynamics. Hydric stress induces osmotic adjustment through solute accumulation (including sugars and organic acids), enhancing cellular integrity while improving fruit palatability.

Furthermore, tillage operations modify plant water acquisition patterns while simultaneously regulating soil thermal regimes. Empirical evidence demonstrates that elevated growth temperatures negatively influence organic acid biosynthesis in fruit crops (e.g., banana [*Musa spp.*], grape) (Arias et al. 2021; Somkuwar and Dhole 2025). Thermal stress disrupts enzymatic functionality in glycolysis and TCA cycle pathways, suppressing organic acid production. Concurrently, membrane lipid peroxidation under heat stress increases cytoplasmic leakage of organic acids (Farber et al. 2023), thereby reducing fruit titratable acidity. Consequently, superficial tillage in sandy-textured apple/pear orchards disrupts evaporative soil channels, achieving 2.8–4.3 °C thermal mitigation through enhanced latent heat dissipation.

### Fertilization practices: their impact on malate accumulation dynamics in horticultural crops

As premium economic commodities, horticultural cultivars are required to attain dual optimization of yield and quality parameters at harvest maturity. Naturally occurring plant phenotypes, however, consistently demonstrate suboptimal productivity and inferior organoleptic characteristics. This productivity-quality paradox has driven the development of targeted fertilizer formulations-including organic (OFA) and mineral (MFA) nutritional amendments-to synergistically enhance both agronomic output and fruit biochemical profiles.

Organic fertilizer application to *Citrus* spp. enhances total biomass production while positively regulating carbohydrate partitioning within plant tissues (Martinez-Alcantara et al. 2016). These amendments further ameliorate soil architecture and elevate soil organic carbon stocks. Fruit from organically fertilized trees demonstrate superior quality attributes, characterized by enriched sugar-organic acid profiles and extended postharvest longevity. While organic treatments show negligible impacts on fruit sugar content or sweetness indices, they significantly elevate organic acid (malate and citrate) concentrations (Jasminka et al. 2025).

Contrastingly, potassium fertilization in *Vitis vinifera* L. stimulates grape sugar accumulation while suppressing organic acids (tartaric, malic, citric acids) (Wang et al. 2024a, b). Cellular K^+^ excess triggers root biosynthesis of malate and citrate anions to counter ionic toxicit (Lopez-Bucio et al. 2000), a mechanism corroborated in *Malus domestica* ‘Red Delicious’ where potassium inputs reduced fruit malate/citrate levels by 150 days post-anthesis.

Nitrogen management studies reveal moderate application during fruit expansion optimizes quality, whereas persistent high-N conditions during maturation elevate malate retention (+ 18–22%) while depressing soluble sugars (−14–19%), ultimately diminishing sensory quality. Notably, rice (*Oryza sativa*) exhibits enhanced growth under organic acid-K formulations versus KCl, with small-molecule organic-K specifically upregulating TCA cycle intermediates (malate/citrate) unavailable to chloride-based fertilizer (Liu et al. 2023). Phosphorus deficiency in Gossypium hirsutum suppresses fiber elongation by repressing the expression of *PM H*^+^*-ATPase* (−37%), *VH*^+^*-ATPase* (−29%), *VH*^+^*-PPase* (−42%), and *PEPC* (−33%), thereby disrupting malate synthesis/transport and compromising cellular osmotic regulation (Sun et al. 2023).

## Concluding remarks

This comprehensive review systematically covers multiple dimensions of malate biology, encompassing its metabolic pathways, transmembrane transport mechanisms, regulatory networks, evolutionary trajectories of associated genes, and the impact of agricultural management on malate accumulation. Accumulating evidence indicates that malate accumulation is predominantly regulated by the activity of malate transport proteins, positioning these molecular transporters as pivotal determinants in horticultural crop improvement strategies. The analysis further reveals an intriguing evolutionary pattern where genetic domestication of malate-associated loci preceded sugar-related selection in key horticultural species such as apple. Notably, Ma1 has undergone significant artificial selection during apple domestication, underscoring its critical role in malate accumulation. From an agronomic perspective, environmental variables including thermal conditions, photoperiod regulation, irrigation management, and soil nutritional composition exert substantial influence on malate metabolic dynamics.

While molecular investigations have identified several malate-associated genetic components, several critical gaps remain to be addressed: (1) The spatiotemporal expression patterns of related genes affecting the dynamics of malate including biosynthesis enzymes (e.g., *NAD-MDH*, *PEPC*), transporters (e.g., *ALMT* family), and transcription factors (e.g., *MdMYB1*, *MdbHLH3*) during growth and development, along with the mechanisms underlying certain specific expression patterns have not yet fully understood. Especially, elucidating the expression characteristics of relevant factors in the dynamic process of fruit development is significant for understanding the metabolic dynamics of malic acid and the formation of fruit quality. (2) The mechanism of epigenetic regulation in malic acid metabolism and transport still needs further investigation. Recent studies have revealed that DNA methylation participates in the metabolic process of pear fruit by modulating related genes, thereby having crucial effects on fruit development and ripening (Gu et al. 2024). Moreover, MdH1.1, a potential epigenetic modifier, plays a significant role in sorbitol-modulated malate accumulation (Hu et al. 2025). These findings collectively suggest that epigenetic regulation probably plays a pivotal role in the dynamic control of malate metabolism. (3) The molecular mechanisms underlying the interaction between environmental and genetic factors in regulating the dynamic balance of malate needs further exploration. For example, the integrated impact of multiple environmental factors on the dynamics of malate has not yet been elucidated. Moreover, the selective signatures of malate metabolic regulatory elements during climate-adaptive evolution also needs further exploration. In addition, the interaction between rhizosphere microbiota and malate will contribute to a deeper understanding of the combined effects of environmental factors and genotypes on malate dynamics.

The above unresolved aspects provide promising research directions for optimizing fruit quality through targeted metabolic engineering and precision cultivation practices. Firstly, with the development of omics technologies and CRISPR-Cas9 gene editing, the era of whole-genome molecular design breeding for fruit trees is approaching. By integrating multi-omics approaches, more and more candidate genes involved in malate metabolism will be efficiently identified at the whole-genome level, which will greatly facilitate the systematic decoding of malate regulatory network at multi-dimensional scale. And excellent germplasm resources with appropriate malate level will be cultivated using whole-genome molecular design breeding. Secondly, the gene-environment-phenotype continuum can be established through integrated computational models that synthesize varietal genomes, epigenetic landscapes, and real-time environmental data streams from IoT-enabled smart orchards. Machine learning approaches could decode the nonlinear relationships between cultivation parameters (microclimate modulation, nano-fertilization, deficit irrigation) and malate biosynthetic rhythms. Thirdly, comparative pan genomic analysis across apple cultivars and their wild relatives may reveal conserved modules in malate evolution, providing powerful information for targeted reactivation of ancestral metabolic pathways. Combining synthetic biology, related research will guide the development of climate-adaptive apple cultivars with optimized acid-sugar balance. Such advances will ultimately facilitate the design of customized fruit production systems adapted to evolving market and climatic demands.

## Supplementary Information

Supplementary Material 1

## Data Availability

The datasets used and/or analysed during the current study are available from the corresponding author on reasonable request.

## Abbreviations

1-MCP: 1-Methylcyclopropene
ABA: Abscisic acid
ALMT: Aluminumactivated malate transporter
AS: Alternative splicing
bHLH: Basic helix-loop-helix
BT2: BTB-BACK-TAZ scaffold protein 2
COP1: Constitutively photomorphogenic 1
ERF: Ethylene response factor
GWAS: Genome-wide association study
H3K27me3: Trimethylation of histone H3 at lysine 27
H3K4me3: Trimethylation of histone H3 at lysine 4
HXK1: Hexokinase 1
MBW: MYB-bHLH-WD40
MDH: Malate dehydrogenase
MFA: Mineral fertilizer activator
MYB: R2R3-MYB transcription factor
NAC: NAMATAF1/2-CUC2
OAA: Oxaloacetate
OFA: Organic fertilizer activator
PEP: Phosphoenolpyruvate
PEPC: Phosphoenolpyruvate carboxylase
PEPCK: Phosphoenolpyruvate carboxykinase
pH: Potential of hydrogen
PH5: P-type ATPase 5
PP2CH: Protein phosphatase 2CH subfamily
PTMs: Post-translational modifications
PUB29: Plant U-box E3 ubiquitin ligase 29
QTL: Quantitative trait loci
SIZ1: Sap and miz1 domain containing ligase1
SNP: Single nucleotide polymorphism
TCA: Tricarboxylic acid
tDT: Tonoplast dicarboxylate transporter
TFM6: Tomato fruit malate on chromosome 6
TFs: Transcription factors
VHA-A: Vacuolar ATPase subunit A
VHP1: Vacuolar pyrophosphatase 1
WRKY: WRKY transcription factor
