# Supplementary material for: Malate metabolism in horticultural crops: mechanistic insights and agricultural practices for quality improvement
Source: Mol Hortic. 2025 Oct 23;5:58. doi: 10.1186/s43897-025-00196-6 (PMC12548192; doi:10.1186/s43897-025-00196-6)
Supplement: Supplementary file 1 — Supplementary Material 1 [file 43897_2025_196_MOESM1_ESM.docx]

**Table S1.** **Table of sequence number and gene name groupings with chromosome distribution.**

| **Sequence number** | **Gene name** | **Group** | **Chromosome** |
| --- | --- | --- | --- |
| AT1G08430.1 | AtALMT1 | D | 1 |
| AT1G08440.1 | AtALMT2 | A | 1 |
| AT1G18420.1 | AtALMT3 | F | 1 |
| AT1G25480.1 | AtALMT4 | F | 1 |
| AT1G68600.1 | AtALMT5 | F | 1 |
| AT2G17470.2 | AtALMT6 | F | 2 |
| AT2G27240.1 | AtALMT7 | A | 2 |
| AT3G11680.2 | AtALMT8 | B | 3 |
| AT3G18440.1 | AtALMT9 | E | 3 |
| AT4G00910.1 | AtALMT10 | C | 4 |
| AT4G17970.1 | AtALMT12 | D | 4 |
| AT5G46600.1 | AtALMT13 | D | 5 |
| AT5G46610.1 | AtALMT14 | D | 5 |
| Cs_ont_5g004830 | CsALMT10-2 | C | 5 |
| Cs_ont_5g010080 | CsALMT8-1 | H | 5 |
| Cs_ont_5g036920 | CsALMT9 | E | 5 |
| Cs_ont_5g048790 | CsALMT9L | H | 5 |
| Cs_ont_5g050080 | CsALMT10-1 | C | 5 |
| Cs_ont_6g009470 | CsALMT8-2 | B | 6 |
| Cs_ont_7g011370 | CsALMT12 | D | 7 |
| Cs_ont_7g019590 | CsALMT4 | F | 7 |
| Cs_ont_8g009120 | CsALMT2L-3 | C | 8 |
| Cs_ont_8g009150 | CsALMT2L-2 | A | 8 |
| Cs_ont_8g009160 | CsALMT2 | A | 8 |
| Cs_ont_8g009180 | CsALMT2L-1 | A | 8 |
| MD00G1017600 | MdALMT2L-5 | G | 0 |
| MD00G1049200 | MdALMT2L-6 | C | 0 |
| MD03G1155200 | MdALMT2L-3 | G | 3 |
| MD03G1155400 | MdALMT2L-1 | C | 3 |
| MD03G1155500 | MdALMT2L-4 | B | 3 |
| MD03G1266500 | MdALMT13L-1 | H | 3 |
| MD06G1032000 | MdALMT12L-1 | D | 6 |
| MD06G1096000 | MdALMT9L-2 | H | 6 |
| MD06G1114500 | MdALMT10L-2 | C | 6 |
| MD06G1214800 | MdALMT9-1 | E | 6 |
| MD07G1153600 | MdALMT10 | B | 7 |
| MD11G1173000 | MdALMT2L-2 | C | 11 |
| MD11G1287000 | MdALMT13L-2 | H | 11 |
| MD11G1287100 | MdALMT12L-3 | H | 11 |
| MD12G1040500 | MdALMT8L-2 | A | 12 |
| MD13G1044200 | MdALMT4L-2 | E | 13 |
| MD13G1044400 | MdALMT4L | E | 13 |
| MD14G1039500 | MdALMT8L-1 | A | 14 |
| MD14G1116200 | MdALMT9L-1 | H | 14 |
| MD14G1135700 | MdALMT10L-1 | C | 14 |
| MD14G1135900 | MdALMT10L-3 | C | 14 |
| MD14G1225700 | MdALMT9-2 | E | 14 |
| MD16G1045000 | MdALMT4L-1 | F | 16 |
| MD16G1045200 | MdALMT4 | F | 16 |
| MD16G1276700 | MdALMT12L-2 | D | 16 |
| VIT_202s0012g01030.1 | VvALMT12-1 | D | 2 |
| VIT_202s0025g00700.1 | VvALMT9-1 | H | 2 |
| VIT_202s0025g00700.2 | VvALMT4-1 | H | 2 |
| VIT_202s0025g00700.3 | VvALMT9-2 | H | 2 |
| VIT_202s0025g02320.1 | VvALMT9-5 | C | 2 |
| VIT_202s0025g02320.2 | VvALMT10-1 | C | 2 |
| VIT_202s0025g02320.3 | VvALMT10-3 | C | 2 |
| VIT_206s0009g00450.1 | VvALMT2-1 | A | 6 |
| VIT_206s0009g00480.1 | VvALMT2-2 | B | 6 |
| VIT_206s0080g00170.1 | VvALMT2-3 | B | 6 |
| VIT_206s0080g00200.1 | VvALMT8-2 | C | 6 |
| VIT_207s0104g00100.1 | VvALMT12-2 | H | 7 |
| VIT_207s0104g00100.2 | VvALMT12-5 | H | 7 |
| VIT_207s0104g00100.3 | VvALMT12-3 | H | 7 |
| VIT_207s0104g00100.4 | VvALMT12-4 | H | 7 |
| VIT_207s0104g00100.5 | VvALMT12-6 | H | 7 |
| VIT_208s0105g00250.1 | VvALMT8-1 | A | 8 |
| VIT_215s0046g00130.1 | VvALMT10-2 | C | 15 |
| VIT_217s0000g03850.1 | VvALMT9-3 | F | 17 |
| VIT_217s0000g03850.2 | VvALMT9-4 | F | 17 |
| VIT_218s0122g00020.1 | VvALMT4-2 | F | 18 |

**Table S2.** **Details of the genome sequences, gene sequence, and protein sequences used in this study.**

| **Species** | **Genome Sequence** | **Gene sequence** | **Protein sequence** |
| --- | --- | --- | --- |
| *Arabidopsis thaliana* | Athaliana_447_TAIR10.fa.gz | Athaliana_447_Araport11.gene.gff3.gz | Athaliana_447_Araport11.protein.fa |
| *Malus domestica* | GDDH13_1-1_formatted.fasta.bz2 | gene_models_20170612.gff3.bz2 | GDDH13_1-1_prot.fasta.bz2 |
| *Citrus sinensis* | SWO.v3.0.genome.fa.gz | SWO.v3.0.gene.fa.gz | SWO.v3.0.protein.fa.gz |
| *Vitis vinifera* | Vvinifera_457_Genoscope.12X.fa.gz | Vvinifera_457_v2.1.gene.gff3.gz | Vvinifera_457_v2.1.protein.fa.gz |
